# Physiological and Biochemical Responses of four cassava cultivars to drought stress

**DOI:** 10.1038/s41598-020-63809-8

**Published:** 2020-04-24

**Authors:** Yanmei Zhu, Xinglu Luo, Gul Nawaz, Jingjing Yin, Jingni Yang

**Affiliations:** 1 Agricultural College of Guangxi University, Nanning, 530005 China; 2 State Key Laboratory for Conservation and Utilization of Subtropical Agro-bioresources, Nanning, 530005 China

**Keywords:** Plant physiology, Plant stress responses

## Abstract

The antioxidant mechanism is crucial for resisting oxidative damage induced by drought stress in plants. Different antioxidant mechanisms may contribute to the tolerance of cassava to drought stress, but for a specific genotype, the response is still unknown. The objective of this study was to investigate antioxidant response and physiological changes of four cassava genotypes under water stress conditions, by keeping the soil moisture content as 80% (control), 50% (medium), 20% (severe) of field capacity for a week. Genotypes RS01 and SC124 were keeping higher relative water content (RWC) and relative chlorophyll content (SPAD value) and less affected by oxidative stress than SC205 and GR4 under drought stress. RS01 just showed slight membrane damage and oxidative stress even under severe drought conditions. A principal component analysis showed that cassava plant water status was closely related to the antioxidant mechanism. Antioxidant response in genotypes RS01 and SC124 under drought stress might attribute to the increased accumulation of ascorbate (AsA) and glutathione (GSH) content and higher superoxide dismutase (SOD) and catalase (CAT) activities, which explained by the up-regulation of *Mn-SOD* and *CAT* genes. However, Genotypes SC205 and GR4 mainly depended on the accumulation of total phenolics (TP) and increased glutathione reductase (GR) activity, which attribute to the up-regulation of the *GR* gene. Our findings could provide vital knowledge for refining the tactics of cultivation and molecular breeding with drought avoidance in cassava.

## Introduction

Drought stress is commonly induced by rainfall patterns, greenhouse effect and the variations of temperature. It is an important environmental stress factor that limits plant growth, regulation, and distribution^1–3^. Compared with other abiotic stresses, drought stress exerts more restrictions on crop productivity^4^, especially on the marginal lands with poor soils and limited water resources. For resource-limited small farmers in these marginal areas, cassava (*Manihot esculenta* Crantz) is an important staple food crop due to its inherent tolerance to stressful environments^5^. Because of its starchy roots, cassava is used for starch extraction and as feed resource and feedstock production in China and other Southeast Asian countries^6^. Drought is one of the main constraints that limit cassava growth and production, particularly during the first three months after planting^7^. Therefore, it is urgent to understand the mechanisms underlying drought tolerance of cassava at the seedling stage.

Plants have developed defense mechanisms which enable them to adapt and survive under drought condition in their life cycle^8^. The drought response of plants varies from species and the severity of the drought stress. The mechanisms of cassava resistant to water deficit include stomatal closure, decreased leaf area, the proper maintenance of net photosynthetic rate for prolonged drought, and the ability to explore water from deep soil layers^9^. The defense strategies against drought environment also vary from different cassava cultivars. During a mild drought period, SC124 cassava cultivar showed a “survival” mode by early stomatal closure and decreased photosynthesis resulting in early growth quiescence, while shedding of older leaves but continuing to grow in Agr7 cassava cultivar^10^. The strategy of SC124 cultivar is more beneficial for survival under severe drought stress than Agr7.

During a prolonged drought stress condition, reactive oxygen species (ROS) generate excessively and cause oxidative damage^11^. ROS can damage multiple cellular components such as proteins and lipids, and unlimited disruption will finally lead to cell death^12,13^. In order to counteract the production of ROS under such conditions, antioxidant defense mechanisms were formed in the long-term evolvement in plants. One vital member of this defense system is enzymatic machinery including superoxide dismutase (SOD), ascorbate peroxidase (APX), peroxidase (POD), glutathione reductase (GR), catalase (CAT), etc. In addition, non-enzymatic antioxidants such as ascorbic acid (AsA), glutathione (GSH), total phenolics (TP) and total flavonoids (TF) also contribute to the alleviation of oxidative damage. The activities of SOD, CAT, and POD in cassava leaves increased to remove superoxide free radicals and control the level of membrane lipid peroxidation during drought stress conditions^14^. However, mechanisms of non-enzymatic antioxidants in cassava under water deficit are still unknown.

Currently, it is prevalent to study the mechanism of stress response at genetic, physiological and molecular levels^15^. To have a better understanding of the factors affecting antioxidant regulation, it is important to associate antioxidant enzyme activity and related-gene expression in different genotype species^16^. However, the studies on response mechanism under drought stress in cassava were limited to the physiological or molecular method only. Taking this into account, the present research was aimed at the elucidation of antioxidant response mechanism under drought in cassava seedlings combining genetic, physiological and molecular approaches.

## Results

### Relative water content and SPAD values

**Figure 1:**
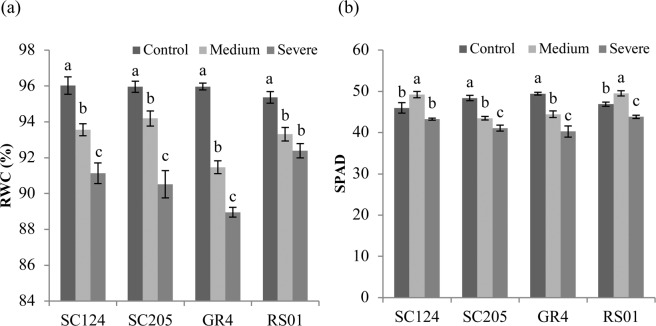
Physiological parameters in four cassava genotypes under control, medium, and severe stress. Leaf concentrations of (**a**) relative water content (RWC), (**b**) chlorophyll content (SPAD), are shown as means with SD (n = 4). For each genotype, different letters above the bars indicate significant differences between treatments.

Drought stress caused a significant decline in the RWC of each genotype. The reduction of RWC in GR4 was more obvious than other genotypes (Fig. 1(a)). When severe drought stress occurred, RWC of GR4 was reduced by 7.31%, compared with 5.10%, 5.67% and 3.12% in SC124, SC205, and RS01 respectively. Changes in SPAD values varied in different genotypes (Fig. 1(b)). In genotypes SC205 and RS01, a stress-dependent reduction of SPAD values with increasing drought intensity was observed. In contrast, the SPAD values of SC124 and RS01 shoots increased under medium drought stress while decreased after exposure to severe drought stress. RWC and SPAD of RS01 and SC124 were both higher than that of GR4 and SC205 under severe drought stress. The results of two-way ANOVA revealed the significant differences for both RWC and SPAD levels, regarding treatments, genotypes and their interactions (Table 1).

**Table 1:** Drought treatments of the chosen cassava genotypes.

Soure of variance	DF	RWC	SPAD	RLC	MDA	H_2_O_2_	AsA	GSH	TP	TF	SOD	POD	CAT	APX	GR
Genotype (G)	3	142.49**	62.21**	52.44**	11.35**	123.26**	318.49**	69.92**	45.35**	199.92**	268.61**	101.80**	138.90**	0.63^NS^	126.65**
Treatment (T)	2	9.02**	6.99**	28.97**	5.44**	21.26**	83.55**	145.92**	13.25**	15.50**	9.44**	10.64**	74.79**	242.68**	43.63**
G × T	6	5.12**	9.85**	12.02**	0.73^NS^	10.50**	48.02**	54.03**	5.14**	8.54**	32.15**	17.54**	26.86**	47.25**	25.64**

Results of two-way ANOVAs for the independent variables ‘genotype’ and ‘treatment’, and the ‘genotype × treatment’ interactions. The measurement included relative water content (RWC), chlorophyll content (SPAD), relative leaf conductivity (RLC), malondialdehyde (MDA), hydrogen peroxide (H_2_O_2_), ascorbate (AsA), glutathione (GSH), total phenolics (TP), total flavonoids (TF), specific activities of superoxide dismutase (SOD), peroxidase (POD), catalase (CAT), ascorbate peroxidase (APX), glutathione reductase (GR). DF:Degrees of freedom, NS:non-significant, *significant at *P* = 0.05, **significant at *P* = 0.01

### Relative leaf electrical conductivity (RLC), malondialdehyde (MDA) and hydrogen peroxide (H_2_O_2_) content

**Figure 2:**
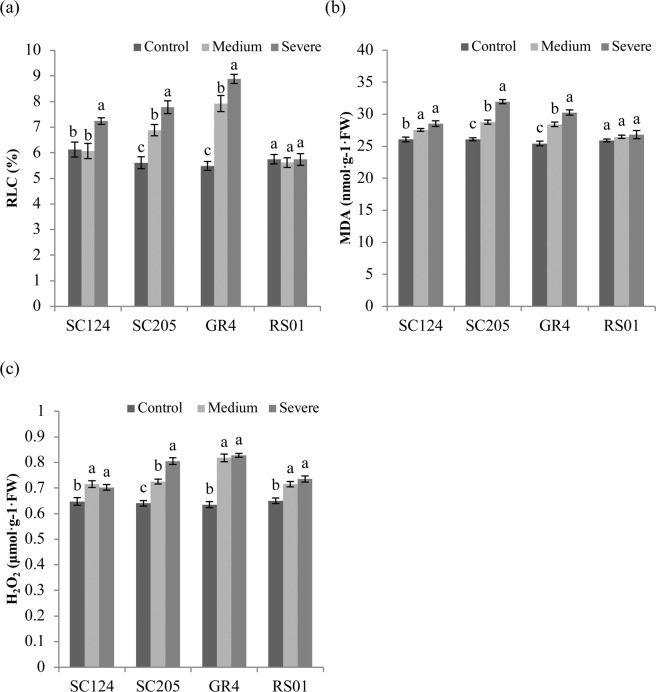
Oxidative damage markers in four cassava genotypes under control, medium, and severe stress. Leaf concentrations of (**a**) relative leaf conductivity (RLC), (**b**) malondialdehyde (MDA), (**c**) hydrogen peroxide (H_2_O_2_), are shown as means with SD (n = 4). For each genotype, different letters above the bars indicate significant differences between treatments.

Drought stress has a significant effect on RLC and MDA of all genotype shoots except RS01(Fig. 2(a,b)). In genotypes SC205 and GR4, RLC and MDA levels increased significantly with increasing drought stress. However, unclear correlations with drought intensity were showed in both RLC and MDA levels of SC124. H_2_O_2_ accumulations of all genotypes tended to rise under drought stress conditions (Fig. 2(c)). The greatest increase was up to 30.31% in GR4, followed by 25.78%, 13.08% and 10.42% in SC205, RS01, and SC124, respectively. MDA and H_2_O_2_ of RS01 and SC124 were both higher than that of GR4 and SC205 under severe drought stress. All the analyzed parameters (RLC, MDA, and H_2_O_2_) were significantly affected by treatments, genotypes, and their interactions, except for the interaction between the two factors as for MDA (Table 1).

### Non-enzymatic antioxidants

**Figure 3:**
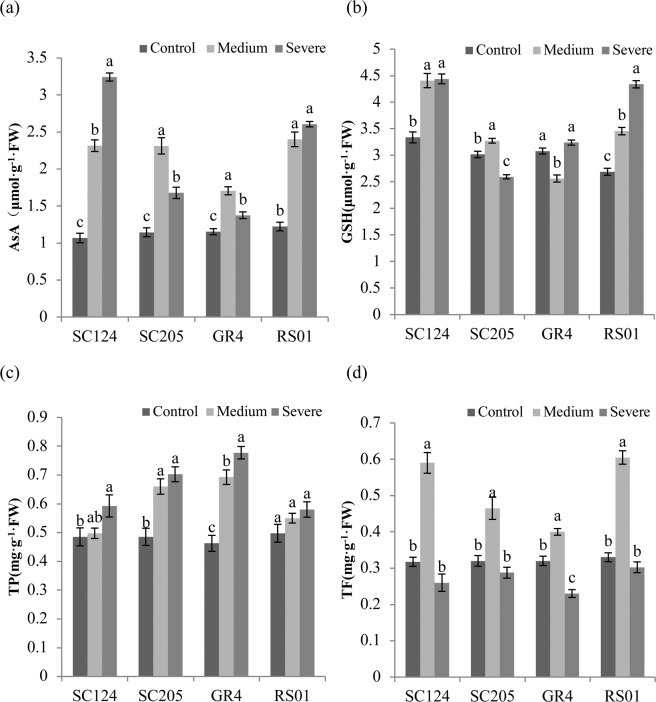
Non-enzymatic antioxidants in four cassava genotypes under control, medium, and severe stress. Leaf concentrations of (**a**) ascorbate (AsA), (**b**) glutathione (GSH), (**c**) total phenolics (TP), **(d**) total flavonoids (TF), are shown as means with SD (n = 4). For each genotype, different letters above the bars indicate significant differences between treatments.

AsA content was similar in the four cassava genotypes under normal condition and increased significantly after exposure to medium drought stress (Fig. 3(a)). The strongest response to drought was showed in the AsA content of SC124, which was induced almost 3-fold under severe drought stress. Change patterns of GSH content varied from different cassava genotypes under water stress (Fig. 3(b)). The strongest drought-induced increase in GSH content was observed in RS01 under severe drought stress. TP content of all genotypes increased in parallel with increasing drought stress with the greatest increase seen in GR4 (68.11%), followed by SC205 (44.85%), SC124 (22.16%) and RS01 (16.58%), respectively (Fig. 3(c)). After exposure to drought stress, all the genotypes showed similar changing patterns of TF content with the maximum level seen under medium drought (Fig. 3(d)). Two-way ANOVA showed that all compounds analyzed (AsA, GSH, TP, and TF) differed significantly in respect of treatments, genotypes and their interactions (Table 1).

### Antioxidant enzyme activities

**Figure 4:**
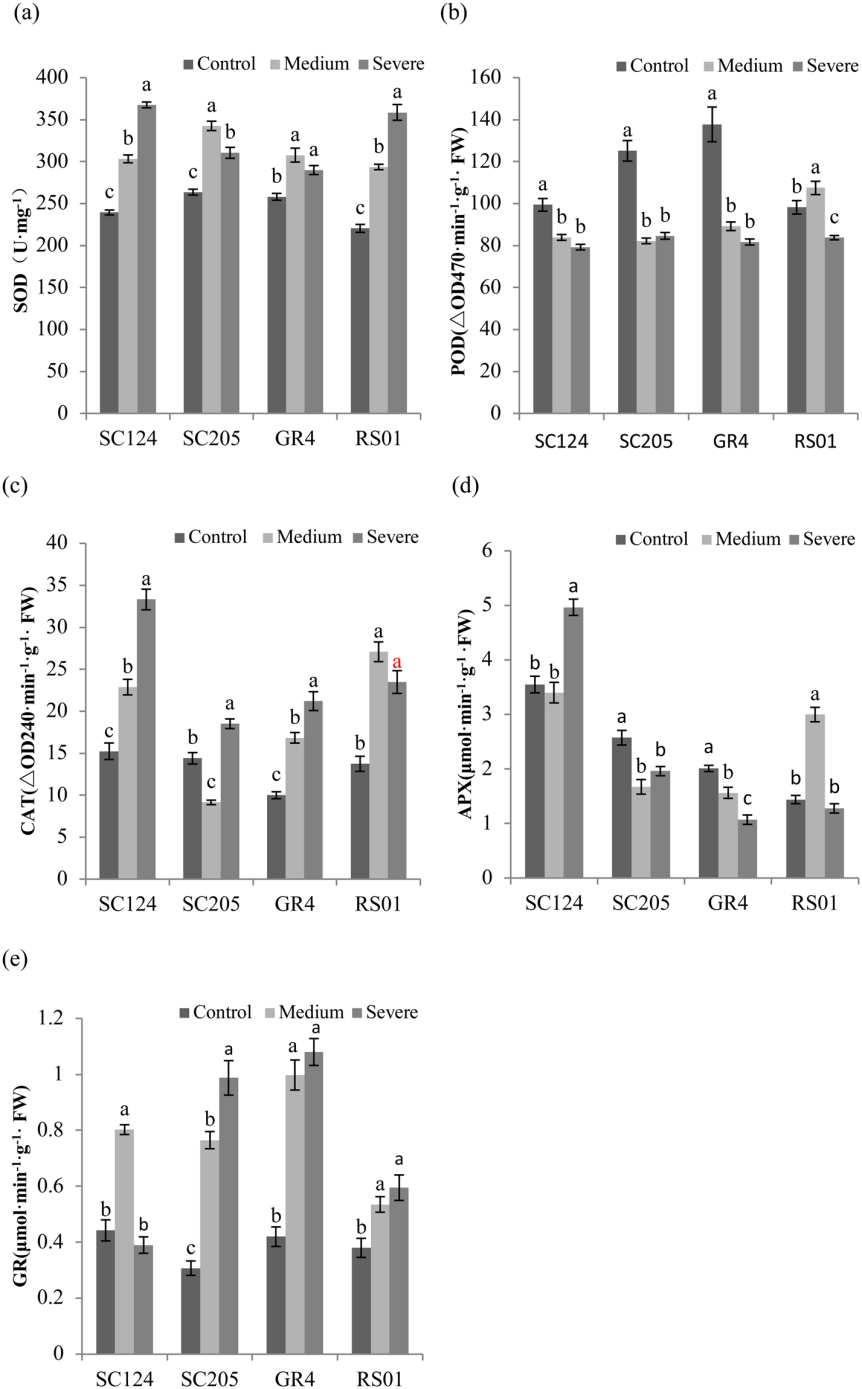
The activity of antioxidant enzymes in four cassava genotypes under control, medium, and severe stress. The graphs show specific activity of (**a**) superoxide dismutase (SOD), **(b**) peroxidase (POD), (**c**) catalase (CAT), (**d**) ascorbate peroxidase (APX), (**e**) glutathione reductase (GR), as means with SD (n = 4). For each genotype, different letters above the bars indicate significant differences between treatments.

Variation patterns of antioxidant enzyme activities in response to drought stress were altered depending on genotypes and enzymes. SOD activity rose in drought-treated plants of each genotype as compared with control (Fig. 4(a)). This increase was most obvious in RS01 (62.69% of control), followed by SC124 (53.56%), SC205 (17.75%) and GR4 (12.34%), respectively. POD activity tended to decline under drought in each genotype although the trend was not suitable for RS01 where activity increased under medium drought (Fig. 4(b)). CAT activity of each genotype tended to rise under drought stress and the stronger increase was shown in genotypes SC124 and RS01 (Fig. 4(c)). The APX activities of SC205 and GR4 declined in response to drought while an increase of this enzyme activity was seen in RS01 and SC204 induced by medium and severe drought, respectively (Fig. 4(d)). Drought stress caused an increase in GR activity of each genotype and this increase was greater in SC205 and GR4 (Fig. 4(e)). All enzymatic activities were significantly influenced by treatments, genotypes, and their interactions. An exception was for the differences of APX activity between treatments, which was not significant (Table 1).

### Gene expression in response to drought stress

**Figure 5:**
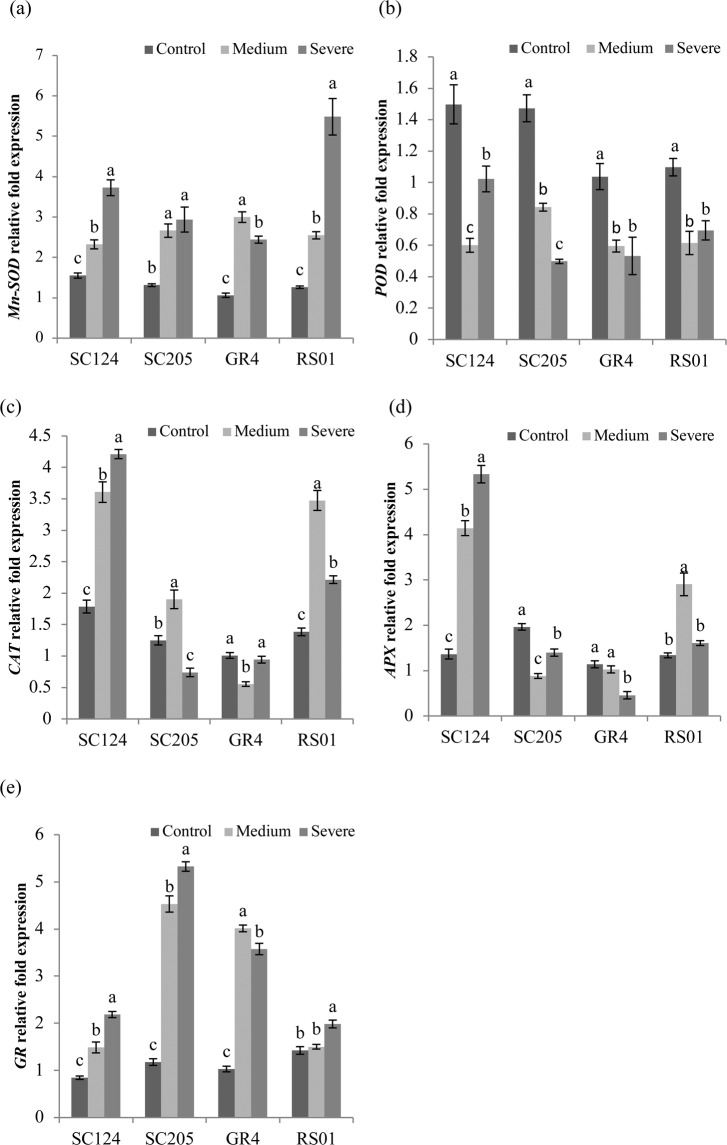
Antioxidant enzyme-related genes expression in four cassava genotypes under control, medium, and severe stress. The graphs show specific gene of (**a**) mitochondrial manganese superoxide dismutase (*Mn-SOD*), (**b**) peroxidase (*POD*), (**c**) catalase (*CAT*), (**d**) ascorbate peroxidase (*APX*), (**e**) glutathione reductase (*GR*), as means with SD (n = 4). For each genotype, different letters above the bars indicate significant differences between treatments.

The expression analysis of five genes related to antioxidant enzymes was conducted by using specific primers (Table 2). These genes include *Mn-SOD* (encoding mitochondrial manganese superoxide dismutase), *POD* (peroxidase), *CAT* (catalase), *APX* (ascorbate peroxidase) and *GR* (glutathione reductase). In response to water stress, the expression of *Mn-SOD* of each genotype was upregulated as compared to corresponding control with the highest increase in transcript level seen in RS01(almost up to 6-fold) under severe drought stress (Fig. 5(a)). The expression of *Mn-SOD* was in line with the SOD activity change. The transcriptional level of *POD* in all genotypes was inhibited by drought. The expression of *POD* was consistent with the change in the profile of POD activity only in GR4 (Fig. 5(b)). The expression of *CAT* tended to be upregulated after exposure to drought in each genotype, except for GR4 and SC205 under medium and severe drought stress, respectively (Fig. 5(c)). The transcript level of *CAT* going along with the CAT activity change was only observed in SC124 and RS01 genotypes. The expression level of APX was upregulated in genotypes SC124 and RS01 whilst downregulated in SC205 and GR4 (Fig. 5(d)). The trend of changes in the expression of APX was similar to the APX activity variation. Drought stress induced the up-regulation of the *GR* gene in each genotype (Fig. 5(e)). The changing trend in the transcript level of GR was similar to the GR activity changes in all genotypes except SC124.

**Table 2:** Primers used for analyzing expression levels of genes related to enzyme activities.

Gene name	Primers
*Mn-SOD*	F: CCCAGCATCATACCACATAGA R: GAGATCAGGGAGCGAGAAAGT
*POD*	F: CTCCGCGATGCTGTCCACAAG R: ACGACACCGTCTCGCCTTCCT
*CAT*	F: GTGGTTCCTGGGATTCACTATTC R:AGGCAGCATCTTGTAGTTGGGT
*APX*	F: AACTTACGACGTGAAGACGAACA R: AACAACACCAGCGAGCTGATAG
*GR*	F: CGATGATGAAATGAGGGCAGTG R: GGGTCCGACCAGTAGCAAAGAG
Actin	F:CGATGGTCGTACAACTGGTAT R: ATCCTCGAATCCAGACACTGT

### Principal component analysis (PCA)

**Figure 6:**
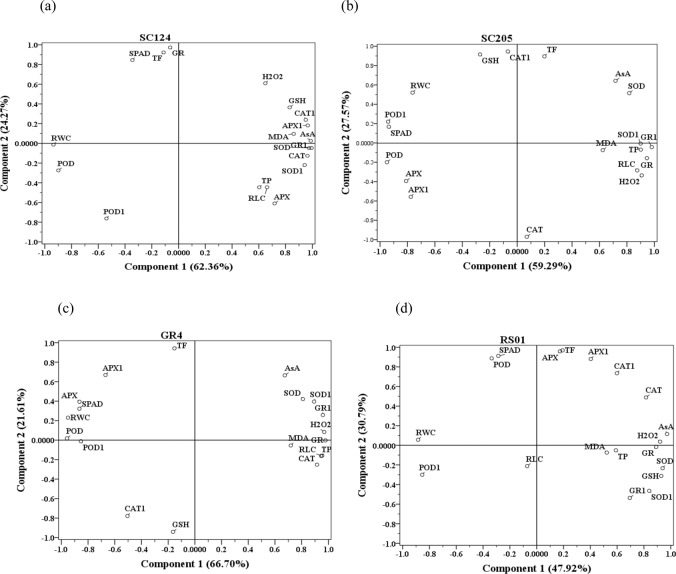
Principal component analysis (PCA). Site score plots of the studied variables in the drought stress treatments, for the four cassava genotypes, SC124 (**a**), SC205 (**b**), GR4 (**c**) and RS01 (**d**). PCA s included these variables: relative water content (RWC), chlorophyll content (SPAD), relative leaf conductivity (RLC), malondialdehyde (MDA), hydrogen peroxide (H2O2), ascorbate (AsA), glutathione (GSH), total phenolics (TP), total flavonoids (TF), specific activities of superoxide dismutase (SOD), peroxidase (POD), catalase (CAT), ascorbate peroxidase (APX), glutathione reductase (GR), and related gene expression of mitochondrial manganese superoxide dismutase (SOD1), peroxidase (POD1), catalase (CAT1), ascorbate peroxidase (APX1), glutathione reductase (GR1).

For each genotype, PCA was performed to investigate correlation existed between physiological, biochemical and transcriptional indexes. Besides, PCA also showed the relationship between leaf water status and antioxidant response mechanism in four cassava genotypes. In genotype SC124, the first and second component explained 62.36% and 24.27% of the data variability, respectively (Fig. 6(a)). According to Pearson correlation coefficients, RCW in SC124 correlated significantly with antioxidant contents(AsA, GSH, and TP), enzymatic activities(SOD, POD, CAT, and APX), gene expression(*Mn-SOD*, *CAT*, *APX1*, and *GR*). For genotype SC205, the first and second components explained 59.29% and 27.57% of the variance, respectively (Fig. 6(b)). RCW in SC205 correlated significantly with TP content, enzymatic activities(POD, CAT, and GR), gene expression(*Mn-SOD*, *POD*, *CAT*, and *GR*).In genotype GR4, 66.70% of the variability was explained by the first component whilst 21.60% by the second component (Fig. 6(c)). RCW in GR4 correlated significantly with TP content, all the enzymatic activities, gene expression (*Mn-SOD, POD*, *APX*, and *GR*). In genotype RS01, 47.92% and 30.79% of variance were explained by the first and second components, respectively (Fig. 6(d)). RWC in RS01correlated significantly with antioxidant contents (AsA, GSH, and TP), enzymatic activities (SOD, CAT, and GR), gene expression (*Mn-SOD*, *POD*, and *GR*). These results suggested that the water status of cassava seedling was closely related to antioxidant response and these relations varied from different genotypes.

## Discussion

As an important environmental limitation, drought has become a rising concern due to its harm to the development and productivity of crop plants^17,18^. Cassava is a major staple food to resource-limited people in marginal areas because of its ability to survive and produce in such poor land with infrequent rainfall and low fertility^10,17,19^. Although cassava is considered as a drought-tolerant crop, its root yield is easily threatened by water stress, especially under serious condition. Therefore, it is critical to screen drought-tolerant cassava germplasm and one of the modern breeding strategies is screening resources for tolerance to severe water deficit during the early period^7^. The present study by using four cassava genotypes can provide a fundamental basis for the identification of drought-tolerant germplasm resources.

Drought stress causes tissue water loss, which results in leaf wilting^20^. As a key factor for estimating plant drought tolerance, RWC is reduced by water deficit and the reduction varies from different genotypes^21^. A similar result was observed in this study. The reduction of RWC under drought was more pronounced in GR4, which showed that the water status in this genotype was more sensitive to drought stress than other genotypes. Water deficit also initiates the degradation of chlorophyll, lead to a decrease of photosynthetic product and eventually inhibit plant growth^22^. Chlorophyll content (SPAD) was frequently used to evaluate plant drought tolerance due to its high correlation with crop yield^23–25^. In the present study, reduction of chlorophyll content (SPAD) induced by drought was strongest in genotype GR4 while minimum in RS01, which is in accordance with previous findings of decreased levels of chlorophyll under drought stress in different plant species^26,27^.

RLC changes can reflect the degree of membrane damage in response to drought. Water deficit increased RLC in genotypes GR4, SC205 and SC124. However, no significant effect of drought on RLC was found in RS01, which showed that membrane injuries of this genotype were slight. MDA is usually used as a reliable marker to judge oxidative stress^28^. Drought stress significantly increased the MDA content of genotypes SC205 and GR4 whilst had no effect on RS01. Therefore, for RS01, the oxidative stress induced by drought was slight at the seedling stage. H_2_O_2_, as one crucial member of ROS induced by drought, increased significantly in the drought-affected cassava seedlings and this increase was stronger in genotypes SC205 and GR4 than RS01 and SC124. In a previous report, Wang *et al*.^29^ showed that the H_2_O_2_ concentration of apple plants was enhanced under drought stress and the greater increase was observed in the sensitive species, which is consistent with our findings.

Non-enzymatic mechanism plays a vital role in contracting oxidative stress and improving plant drought-tolerance^30^. Increased AsA and GSH content were induced by water deficiency in rapeseed seedlings^31^. Similarly, drought stress also caused an increase of AsA and GSH content in cassava seedlings except for unclear variation of GSH content in genotypes SC205 and GR4. Greater increases in both AsA and GSH content were observed in genotypes SC124 and RS01, which showed that both genotypes have a more efficient system for the biosynthesis of these antioxidants. In this experiment, drought stress also enhanced TP content in cassava seedlings. This result is supported by previous findings^32^. Genotypes SC205 and GR4 showed a stronger increase in TP content, which may be one key defense mechanism against oxidative stress induced by drought in these two genotypes. TF content significantly increased under medium drought whilst sharply declined after exposure to severe drought. This rapid consumption of TF compounds may be responsible for constraining the accumulation of H_2_O_2_ by cell^33^.

There is little doubt that antioxidant enzymes are another crucial member of defense mechanisms against oxidative damage induced by drought^34^. SOD, as the first frontline defense against oxidative injury, catalyzes the dismutation of O_2_^−^ and generates H_2_O_2_, which is converted to H_2_O and O_2_ by CAT^35^. In this experiment, SOD and CAT activities were activated by water stress in all four cassava genotypes and the rate of increment was higher in RS01 and SC124. These results are supported by the previous report of higher SOD and CAT activities in drought-stressed caper seedlings^36^. Water deficit significantly depressed POD activity in all genotypes and the stronger depression was observed in genotypes GR4 and SC205, which is in line with the findings of a previous study^37^. Research showed that POD activity is related to the water retention of leaves^38^. Thus, the smaller reduction of RWC induced by drought in genotypes RS01 and SC124 may be attributed to the lower decrease of POD activity as compared with GR4 and SC205. APX activity has been reported to activate in bean cultivars, while, in some cases, is unchanged even declined in specific species^39^. For genotypes SC124 and RS01 in this study, an increase in APX activity was detected in some drought-stressed plants while unchanged in other water-deficient plants. Interesting, this enzyme activity was depressed by water stress in both genotypes SC205 and GR4. These results indicated that the changing trend of APX activity under drought was mainly depended on cassava genotypes. The scavenging of H_2_O_2_ mostly attributed to the activating of APX activity^40^. Therefore, the activation of APX activity in genotypes RS01 and SC124 may be responsible for the lower level of H_2_O_2_ under water deficit stress as compared to GR4 and SC205. The function of enzyme GR is catalyzing glutathione disulfide to GSH^21^. GR activity enhanced in all four genotypes after exposure to drought and the greater increase was observed in SC205 and GR4, which corroborated the results of previous reports^36,41,42^.

Genotypic difference in drought tolerance is one reason for the different ability to activate antioxidant defense in plants under severe drought^43^. The different trends of these non-enzymatic and enzymatic antioxidants in four cassava genotypes displayed distinct regulation mechanisms under drought-induced oxidative stress. For genotypes RS01 and SC124, this regulatory mechanism might be mostly attributed to the accumulation of AsA and GSH content and increased activities of SOD and CAT. However, SC205 and GR4 might depend on the accumulation of TP and increased GR activity to resist oxidative damage.

Drought stress can trigger a series of plant regulation, not only including the physiological and biochemical response but also containing the regulation of gene expression^16^. Studying the relationship between gene expression and stress tolerance can provide reliable information on understanding antioxidant gene activation^44^. In the present study, the expression of *Mn-SOD* in all four genotypes was upregulated and was in accordance with the SOD activity change. The highest increase in transcript level was observed in RS01 under severe drought, which showed that *Mn-SOD* might play an essential role in response to water deficit. These results are in line with the previous reports^16,45,46^. The transcriptional level of *POD* was inhibited by drought and was not consistent with the changes of POD activity in all genotypes except for GR4, which revealed that enzyme activity changes were regulated by the post-transcriptional level which in part might result in enzyme inactivation or degradation. In a previous study, Uzilday *et al*.^47^ found that *CAT* gene expression was correlated with CAT activity in cleome Espinosa whilst this correlation was not showed in Cleome gynandra. A similar result was observed in this experiment with the transcript level of *CAT* going along with the CAT activity only in genotypes SC124 and RS01. In general, the expression levels of *APX* and *GR* were correlated with APX and GR activity, respectively.

## Conclusions

The cassava genotypes RS01 and SC124 were keeping higher RWC and relative chlorophyll content and less affected by oxidative stress at the seedling stage under drought stress. RS01 just showed slight membrane injuries and oxidative stress even under severe drought conditions. The water status of cassava plants was closely related to the antioxidant response. Different regulation mechanisms in the four genotypes in response to oxidative damage were shown by the different trends of antioxidant compounds and enzymes. The mechanism in genotypes RS01 and SC124 might mostly attribute to the increased accumulation of AsA and GSH content and higher SOD and CAT activities, which explained by the up-regulation of *Mn-SOD* and *CAT* genes. However, genotypes SC205 and GR4 might depend on the accumulation of TP and increased GR activity, which attributed to the up-regulation of *GR* gene.

## Materials and Methods

### Materials

Four cassava genotypes were used for the current study, viz., SC124, SC205, GR4, and RS01. These genotypes are widely planted in China.

### Experiment design and sampling

The present experiment was conducted in the glasshouse at Guangxi University (GXU). The stem segments of four genotypes were planted into plastic pots (21 cm × 21 cm) on 30^th^ of March 2018. Each pot was filled with the equal potting mixture (soil: sand: ballast at 2:1:1(v/v/v), respectively) before planting. All cassava shoots were well watered with 0.5 L of water every two days before the application of drought treatments. Dehydration stress treatment was imposed after 50 days of planting, at three different levels, i.e., 80% of field capacity (FC) (control), 50% of FC (medium) and 20% of FC (severe). Five replications were maintained for each genotype and treatment of drought stress. The soil water content was monitored using soil moisture measurement (SU-LPC, Beijing) on a daily basis. The third and fourth fully expanded leaves from each plant were collected after 7 days of treatment. All samples were frozen immediately in liquid nitrogen and then stored at −80 °C until this experiment was finished. Fresh leaf samples were also collected for analyzing the moisture content and the relative electrical conductivity.

### The physiological parameters analysis

#### RWC measurement

The RWC was measured according to Barrs and Weatherley^48^. Fresh leaf samples (0.1 g, FW) were soaked for 24 h in deionized water and the turgor weight (TW) was calculated. The samples subsequently were dried at 80 °C to a constant dry weight (DW). The RWC was measured by using the following equation: Leaf RWC (%) = (FW-DW)/(TW-DW) × 100

#### SPAD values

chlorophyll meter (SPAD-502, Minolta, Japan) was used to determine the SPAD values of functional leaves, which can reflect the relative chlorophyll content. The fourth leaf of each plant was chosen for the determination of SPAD values.

#### RLC measurement

The RLC was measured as described by Chen *et al*.^49^. Leaf samples (1.0 g) were cut into about 50 mm^2^ before weighted. The samples then were incubated in 30 mL deionized water for 2 h at room temperature and kept in vacuum for 20 minutes. A conductivity meter (FE30/EL30, Shanghai) was used to measure electrical conductivity (I_1_). The samples subsequently were kept in a boiling water bath for 20 min. After the solution cooled to room temperature, the electrical conductivity (I_2_) was recorded and the RLC was calculated according to the equation: RLC (%) = I_1_/I_2_ × 100

#### MDA content

Leaf samples (0.5 g) were homogenized in 5 mL trichloroacetic acid (TCA, 0.1%). The homogenate was centrifuged at 11000 × g for 20 min. The supernatant was used for measuring the MDA and H_2_O_2_ content. MDA content was assayed by the method of Chu *et al*.^50^. The supernatant (2 mL) was added to 2 mL of 20% TCA containing 0.6% of the thiobarbituric acid (TBA). The solution was boiled for 30 min and then centrifuged at 4 000 × g for 5 min after cooling. The absorbance of the mixture was measured at 450 nm, 532 nm, and 600 nm. MDA content was estimated according to the following equation:

$${\rm{C}}({\rm{MDA}})/\mu {\rm{mol}}\cdot {{\rm{L}}}^{-1}=6.45\times ({\rm{A}}532-{\rm{A}}600)-0.56\times {\rm{A}}450$$

#### H_2_O_2_ content

H_2_O_2_ content was assayed following the method of Alexieva *et al*.^51^. The mixture contained supernatant (0.5 mL), 100 mM potassium phosphate buffer (0.5 mL) and 1 M KI (2 mL). Absorbance at 390 nm was measured after developing the mixture in darkness for 1 h. H_2_O_2_ content was calculated according to a standard curve.

#### Non-enzymatic antioxidants

Leaf samples (0.2 g) were homogenated with 6% meta-phosphoric acid containing 1 mM ethylenediaminetetraacetic acid (EDTA). The homogenate was centrifugated at 11000 × g for 20 min, and the supernatant was used for AsA and GSH content analysis.

AsA content was determined spectrophotometrically at 265 nm and calculated on a standard curve according to the method of Huang *et al*.^52^. GSH content was assayed as described by Hasanuzzaman and Fujita^31^. The reaction was measured at 412 nm and GSH content was calculated on the standard curve with a known concentration of GSH.

Leaf samples (1.0 g) were extracted in hydrochloric acid: methanol (v:v = 1:100). Total phenolic (TP) content was determined according to Blainski *et al*.^53^ with gallic acid used as a standard. The absorbance of the reaction was measured at 760 nm and TP content was calculated according to the standard concentration.

Total flavonoids (TF) were measured as described by Jia *et al*.^54^. Leaf samples (1.0 g) were cut into small pieces and extracted with 100 ml distilled water in a soxhlet extractor for one hour. The absorbance at 510 nm was recorded using catechin as standard.

#### Antioxidant enzyme activities

Leaf sample (0.2 g) were ground in 5 mL ice-cold phosphate buffer (50 mM, pH 7.8) containing 0.1 mM EDTA and 1% polyvinylpy. The homogenate was centrifuged at 11000 × g for 20 min at 4°C and the supernatant was used for analyzing the activities of SOD, POD, CAT, APX, and GR. The SOD activity was estimated adopting the nitroblue tetrazolium (NBT) method following Chu *et al*.^50^. 50 mM phosphate buffer (pH 7.8), 13.0 mM methionine, 10 µM nitroblue tetrazolium (NBT), 0.1 mM EDTA, 0.1 mM riboflavin and 50 µL of enzyme extraction were mixed and the absorbance was recorded at 560 nm. One unit of SOD was defined as the amount of enzyme needed to restrain 50% of NBT. The POD activity was assayed according to the rate of guaiacol oxidation at 470 nm for 3 min^49^. The reaction mixture included 50 mM phosphate buffer (pH 7.0), 28 mM guaiacol, 5 mM H_2_O_2_, and 50 µL of enzyme extraction. The CAT activity was determined following the method of Aebi^55^. The reaction mixture contained 50 mM phosphate buffer (pH 7.0), 12.5 mM H_2_O_2_ and 50 µL of enzyme extraction. The decrease in absorbance was read at 240 nm for 3 min and the activity of CAT was calculated based on the rate of H_2_O_2_ consumption. APX activity was assayed according to oxidation of AsA at 290 nm for 1 min^56^. The reaction mixture contained 50 mM potassium phosphate buffer (pH 7.0), 0.1 mM EDTA, 0.5 mM AsA, 0.1 mM H_2_O_2_, and 100 µL of enzyme extraction. GR activity was measured following the method described by Hasanuzzaman and Fujita^31^. The reaction mixture included 0.1 M potassium phosphate buffer (pH 7.0), 1 mM EDTA, 0.2 mM NADPH, 1 mM GSSG, and 100 µL of enzyme extraction. The decreased absorbance was recorded at 340 nm for 1 min. The activity of GR was calculated basing on the rate of NADPH consumption.

### Quantitative Real-Time PCR (qRT-PCR)

RNA prep Pure Plant Kit (Huayueyang, Beijing) was used for extracting total RNA of cassava seedling. The expression of *Mn-SOD*, *POD*, *CAT*, *APX* and *GR* genes was analyzed by the qRT-PCR. The cassava *Actin* gene was selected as the internal control. The gene-specific primers designed in this experiment were verified according to the melting curve and agarose gel electrophoresis (Table 2). All qRT-PCR experiments were performed with AceQ qPCR SYBR® Green Master Mix (Vazyme, Nanjing) on a Bio-Rad CFX96TM real-time instrument (Bio-Rad, USA). The relative expression of all genes with four replicates for each sample was calculated using 2^−ΔCt^ method^57^.

### Statistical analysis

SPSS (IBM SPSS Statistics 24.0) was used to analyze data, which was shown as the mean ± SE of four replicates and were tested by one-way ANOVA. The differences between the treatments were conducted by Duncan test (*P* < 0.05). The effect of genotype, treatment and their interaction were tested by a two-way ANOVA. In addition, PCA was used to correlate all data measured in this study, independently for each cassava genotype.
